# Cross-species transmission and histopathological variation in specific-pathogen-free minipigs infected with different hepatitis E virus strains

**DOI:** 10.1186/s13567-024-01337-3

**Published:** 2024-07-09

**Authors:** Soontag Jung, Daseul Yeo, Dong-Joo Seo, In-Soo Choi, Changsun Choi

**Affiliations:** 1 Department of Food and Nutrition, College of Biotechnology and Natural Resources, Chung-Ang University, Anseong, Gyeonggi-Do 17546 Republic of Korea; 2 Department of Food and Nutrition, Gwangju University, Gwangju, 61743 Republic of Korea; 3 Developmental and Reproductive Toxicology Research Group, Korea Institute of Toxicology, Deajeon, 34114 Republic of Korea; 4 Department of Infectious Diseases, College of Veterinary Medicine, Konkuk University, 120 Neungdong-ro, Seoul, Gwangjin-gu 05029 Republic of Korea

**Keywords:** Hepatitis E virus, cross-species transmission, extrahepatic manifestations, histopathology

## Abstract

Hepatitis E virus (HEV) is a major cause of viral hepatitis worldwide. Pigs are the natural host of HEV genotype 3 and the main reservoir of HEV. As the host range of HEV genotype 3 expands, the possibility that HEV from various species can be transmitted to humans via pigs is increasing. We investigated the potential cross-species transmission of HEV by infecting minipigs with swine HEV (swHEV), rabbit HEV (rbHEV), and human HEV (huHEV) and examining their histopathological characteristics and distribution in various organs. Fifteen specific-pathogen-free Yucatan minipigs were infected with swHEV, rbHEV, huHEV, or a mock control. In the present study, we analysed faecal shedding, viremia, and serological parameters over a seven-week period. Our results indicated that swHEV exhibited more robust shedding and viremia than non-swHEVs. Only swHEV affected the serological parameters, suggesting strain-specific differences. Histopathological examination revealed distinct patterns in the liver, pancreas, intestine, and lymphoid tissues after infection with each HEV strain. Notably, all three HEVs induced histopathological changes in the pancreas, supporting the association of HEVs with acute pancreatitis. Our results also identified skeletal muscle as a site of HEV antigen presence, suggesting a potential link to myositis. In conclusion, this study provides valuable insights into the infection dynamics of different HEV strains in minipigs, emphasizing the strain-specific variations in virological, serological, and histological parameters. The observed differences in infection kinetics and tissue tropism will contribute to our understanding of HEV pathogenesis and the potential for cross-species transmission.

## Introduction

Hepatitis E virus (HEV), identified as the fifth most common hepatitis-causing virus in the 1980s, is the predominant cause of viral hepatitis globally [1]. HEV has an average incubation period of 40 days and typically results in self-limited acute hepatitis, with the majority of infected patients being asymptomatic or experiencing mild symptoms [2]. Symptomatic hepatitis E patients typically undergo a prodromal phase marked by non-specific symptoms, such as body aches, fever, and nausea, followed by an icteric phase characterized by general hepatitis symptoms, such as dark urine and jaundice [3]. These symptoms are generally resolved within 4–6 weeks, at which point the individual enters the recovery phase. HEV infection can progress beyond the acute phase and potentially results in chronic hepatitis, particularly in immunocompromised patients, such as those with HIV, organ transplant recipients, or elderly individuals [4]. In addition, HEV infection has been reported to induce various extrahepatic symptoms, such as acute pancreatitis—typically considered an extrahepatic manifestation of general hepatitis—as well as neurological, musculoskeletal, hematological, and renal symptoms [5]. Furthermore, in addition to these common features, the symptoms and epidemiology of HEV exhibit distinct characteristics depending on the genotype.

HEV is a positive-sense single-stranded RNA virus with a non-enveloped icosahedral structure; it belongs to the *Hepeviridae family* [6]. HEV also exists in a quasi-enveloped form. While it is present in a non-enveloped form in the environment and feces, it assumes a quasienveloped form in cell culture fluid and the bloodstream [7]. According to a presentation by the International Committee on the Taxonomy of Viruses in 2023, HEV was renamed *Paslahepevirus balayani* and is divided into eight genotypes, 1 to 8 [8]. The genotypes that infect humans are genotypes 1–4 [9]. Specifically, genotypes 1 and 2 infect only humans and are primarily transmitted through waterborne transmission, particularly in developing countries with poor sanitation. Genotypes 1 and 2 contribute to large-scale epidemics in southern Asia, Africa, and Mexico, annually causing approximately 20 million infections, 3.4 million symptomatic cases, 70 000 deaths, and 3000 stillborns worldwide [10]. In these endemic areas, HEV predominantly affects individuals aged 15–35 years. The mortality rate of HEV infection is generally up to 4%, but it is extremely high for pregnant women in their third trimester, exceeding 20% [11]. However, genotypes 3 and 4 are zoonotic and can infect not only humans but also various animals, such as pigs and rabbits [12]. These genotypes tend to be prevalent in developed countries and are typically transmitted through foodborne transmission or the transfusion of contaminated blood. In contrast to genotypes 1 and 2, genotype 3 primarily affects older individuals and shows a male bias [13]. Based on seroprevalence, it is estimated that 68 000 HEV infections occur annually in France, 100 000 in the United Kingdom (UK), and 300 000 in Germany [14]. Although there have not been large outbreaks of infections with HEV genotypes 3 and 4 in developed countries, their seroprevalence has reached notable levels in the United States of America (USA, 21–40%), France (22–52%), and the UK (42%), depending on the region, suggesting a potentially significant burden [15]. Most cases of chronic hepatitis caused by HEV are associated with genotype 3, and these cases can lead to liver cirrhosis or organ failure [4]. Additionally, extrahepatic manifestations of HEV are most commonly reported for genotype 3 [5]. In addition to genotypes 1–4, a single case of chronic HEV infection from genotype 7 occurred in a liver transplant recipient, possibly due to the consumption of camel meat and milk [16].

Recently, the increase in HEV infections in the UK was primarily attributed to the consumption of contaminated pork products [17]. Pigs serve as the natural host for HEV genotype 3, typically exhibiting subclinical infection. A high seroprevalence rate of HEV has been reported among veterinarians and pig industry workers who have frequent contact with pigs, exposing them to the risk of HEV infection [18]. Furthermore, the host range of HEV genotype 3 is expanding continuously. Initially, affecting pigs, primates, and humans, infections were later observed in rats, rodents, rabbits, and dogs, extending further to include domestic animals such as cattle, sheep, horses, and goats [19]. Expansion of the host range for HEV genotype 3 has led to infections in wild animals such as deer and wild boar; HEV infection has even been observed in dolphins. The pig immune system shares more than 80% of its structure and function with the human immune system [20]. Experimental evidence for zoonotic transmission of pig HEV genotype 3 to humans has been reported [21]. During the host expansion of HEV genotype 3, pigs are likely to serve as intermediate hosts and transmit other species-specific genotypes of HEV to humans. Rabbits are also natural hosts for HEV and are among the main reservoirs of HEV genotype 3 [22]. Some research has been conducted on cross-species transmission of rabbit HEV to pigs and porcine HEV to rabbits, but the focus has been on the detection of HEV, and differences in pathogenesis and infection patterns have not been studied [23]. The aim of this study was to substantiate the possibility of cross-species transmission by infecting minipigs with swine HEV (swHEV), rabbit HEV (rbHEV), and human HEV (huHEV). Our study also aimed to comparatively examine the histopathological characteristics and distribution of each strain.

## Materials and methods

### Experimental design

Fifteen eight-week-old specific-pathogen-free (SPF) Yucatan minipigs were randomly divided into four groups: the swHEV-infected (swHEV, *n* = 4), rbHEV-infected (rbHEV, *n* = 4), huHEV-infected (huHEV, *n* = 4), and mock-infected control (mock, *n* = 3) groups. Before HEV inoculation, rectal swabs were collected from all the pigs, and blood was collected from the jugular vein. Pigs in each group were intravenously inoculated with 1 mL of virus inoculum diluted in phosphate-buffered saline (PBS) to a titre of 1 × 10^6^ genome equivalent copies/mL of swHEV (genotype 3 isolated from a pig farm, GenBank No.: MT007930), rbHEV (genotype 3 isolated from a rabbit farm, GenBank No.: KY496200), or huHEV (genotype 3 isolated from the serum of a patient with chronic hepatitis E, GenBank No.: KC618403.1). The mock group was inoculated with the same amount of PBS. Stool and blood samples were collected weekly for seven weeks post-inoculation (wpi). All pigs were euthanized at 7 wpi, and the liver, duodenum, ileum, pancreas, femoral muscle, thymus, tonsils, lymph glands, and spleen were collected for histological analysis. The collected tissue was immediately fixed in 10% neutral buffered formalin. Tissues were prepared by paraffin embedding after fixation for 48 h. All animal experimental methods were approved by the Chung-ang University Institutional Animal Care and Use Committee (Approval number: 2017-00016).

### RNA extraction and reverse transcription quantitative PCR (RT‒qPCR)

Stool samples were suspended in PBS at a ratio of 1:10 and centrifuged at 8000 ×* g* for 20 min, and the supernatants were used for RNA extraction. The blood collected in BD Vacutainer^®^ lithium citrate tubes (Becton, Dickinson, and Company, Franklin Lakes, NJ, USA) was separated into plasma and peripheral blood mononuclear cells (PBMCs) through density gradient centrifugation using Lymphoprep (STEMCELL Technologies, Vancouver, Canada). RNA was extracted from the stool suspension, plasma, and PBMC samples using an RNeasy Mini Kit (Qiagen, Hilden, Germany) according to the manufacturer’s instructions. RT‒qPCR was performed to detect HEV in each sample following a previously published procedure [24]. Briefly, cDNA was synthesized by reverse transcription at 42 °C for 30 min. The cDNA was subjected to RT‒qPCR under the following conditions: initial denaturation at 95 °C for 3 min, 45 cycles of denaturation at 95 °C for 10 s, annealing at 55 °C for 20 s, and extension at 72 °C for 15 s. The primers used were forward (JVHEVF, 5′-GGT GGT TTC TGG GGT GAC-3′), reverse (JVHEVR, 5′-AGG GGT TGG TTG GAT GAA-3′), and probe (JVHEVP, 5′-FAM- TGA TTC TCA GCC CTT CGC-TAMRA-3′) primers.

### Enzyme-linked immunosorbent assay (ELISA)

To demonstrate the plasma levels of interferon-α (IFN-α) and interferon-γ (IFN-γ) and seroconversion, ELISA was performed using a Pig Interferon α, IFN-α ELISA Kit (CSB-E07328p; Cusabio Technology, Houston, TX, USA), a Porcine IFN-gamma ELISA Kit (ES9RB; Thermo Fisher Scientific, Waltham, MA, USA) and an HEV ELISA 4.0v (veterinary) (63,541–096; MP Biomedicals, Irvine, CA, USA). According to the manufacturer’s standard protocol, IFN-α, IFN-γ, and seroconversion were analysed in the four groups. Finally, the absorbance value was read at 450 nm on an Epoch spectrophotometer (BioTek, Winooski, VT, USA). Quantitative analysis was performed using a standard curve. The cut-off value for seroconversion was calculated as 0.5 plus the mean absorbance of the non-reactive control, according to the manufacturer's instructions. A plasma sample showing an absorbance value greater than the cut-off value was considered positive.

### Plasma biochemistry

The plasma levels of alanine aminotransferase (ALT), aspartate aminotransferase (AST), and lipase were measured on the day of collection using International Federation of Clinical Chemistry methods on a 7020 automatic analyser (HITACHI, Tokyo, Japan).

### Histopathology and immunohistochemistry (IHC)

For histopathological examination, tissue slides were stained with haematoxylin and eosin according to standard protocols. For IHC, slides were deparaffinized, rehydrated, and washed with Tris-buffered saline containing 0.05% Tween 20 (TBS-T), and antigen retrieval was performed using proteinase K in Tris–EDTA buffer (1:200) at 37 °C for 20 min. After endogenous peroxidase was quenched with BLOXALL solution (SP-6000; Vector Laboratories, Newark, CA, USA) for 10 min, non-specific immunoreactivity was blocked with 2.5% normal serum in TBS-T for 1 h. Then, a primary anti-HEV antibody (70R-HR003, 1:700; Biosynth, Staad, Switzerland) was added to the sections and incubated for 1 h at room temperature (20–25 °C). After washing three times with TBS-T and the VECTASTAIN^®^ Elite^®^ ABC-HRP Kit, peroxidase (rabbit IgG) (PK-6101, Vector Laboratories) was used as the secondary antibody. Briefly, the sections were incubated with biotinylated goat anti-rabbit IgG for 30 min, washed three times with TBS-T, and incubated with an avidin–biotin–HRP complex for 30 min. To visualize the immunoreaction sites, the sections were incubated with DAB solution (Vector Laboratories), counterstained with Mayer’s hematoxylin, dehydrated, clarified, and sealed using permanent mounting medium under coverslips. The IHC results were analysed using QuPath version 0.4.4 and are expressed as the percentage of stained area relative to the total area.

### Statistical analysis

All the data are expressed as the means ± SDs. Statistical analysis was performed using one-way ANOVA followed by a Bonferroni post hoc correction via the rstatix package in R software 4.3.1. A *p* value < 0.05 was considered to indicate statistical significance.

## Results

### Different HEV strains cause fecal shedding and viremia in SPF minipigs

The detection of HEV in weekly stool, plasma, and PBMC samples from HEV-infected SPF minipigs revealed fecal viral shedding, cell-free viremia, and cell-associated viremia, respectively. The HEV detection rate for each sample is shown in Table 1. In the mock group, no HEV was detected in the samples during the entire experimental period. The swHEV group showed a high detection rate in minipigs, the natural host. In the swHEV group, the fecal swHEV shedding rate was 50% at 3 wpi and 100% at 4 wpi. The rate of detection of cell-free viremia was 25% at 1 and 4 wpi and 50% at 2 wpi. Similarly, the percentages of cell-associated viremia were 50% and 25% at 1 and 4 wpi, respectively. Infection with huHEV (in the huHEV group), which is known to be zoonotic in pigs, had a lower detection rate than infection with swHEV (in the swHEV group), but the pattern was similar. In the huHEV group, at 1 and 4 wpi, the rate of detection of cell-free viremia was 25%, and the rates of detection of cell-associated viremia were 50% and 25%, respectively. Fecal huHEV shedding was detected in 50% of the minipigs at 1 wpi and decreased to 25% at 2 and 4 wpi. Among the three groups, all the samples in the rbHEV group exhibited the lowest detection rate. In the rbHEV group, rbHEV was intermittently detected in plasma and PBMCs. At 1 and 3 wpi, cell-free viremia was detected in 25% of the minipigs, and the percentages of cell-associated viremia were 25% and 50% at 1 and 4 wpi, respectively. Unlike for the other strains, fecal rbHEV shedding was not detected during any of the experiments. In all three groups, no HEV was detected in any of the samples after 5 wpi. These results indicate that HEVs from other species may also infect pigs.

**Table 1:** **Fecal shedding, cell-free and cell-associated viremia in minipigs infected with HEV from different species**

Group	Sample	No. of Positive samples/Total no. tested at indicated wpi							
		0	1	2	3	4	5	6	7
Mock	Plasma	0/3	0/3	0/3	0/3	0/3	0/3	0/3	0/3
	PBMCs	0/3	0/3	0/3	0/3	0/3	0/3	0/3	0/3
	Feces	0/3	0/3	0/3	0/3	0/3	0/3	0/3	0/3
swHEV	Plasma	0/4	1/4	2/4	0/4	1/4	0/4	0/4	0/4
	PBMCs	0/4	2/4	0/4	0/4	1/4	0/4	0/4	0/4
	Feces	0/4	4/4	4/4	2/4	4/4	0/4	0/4	0/4
huHEV	Plasma	0/4	1/4	0/4	0/4	1/4	0/4	0/4	0/4
	PBMCs	0/4	2/4	0/4	0/4	1/4	0/4	0/4	0/4
	Feces	0/4	2/4	1/4	0/4	1/4	0/4	0/4	0/4
rbHEV	Plasma	0/4	1/4	0/4	1/4	0/4	0/4	0/4	0/4
	PBMCs	0/4	1/4	0/4	0/4	2/4	0/4	0/4	0/4
	Feces	0/4	0/4	0/4	0/4	0/4	0/4	0/4	0/4

PBMCs: Peripheral blood mononuclear cells; wpi: weeks post-inoculation

### Only swHEV affects the serological parameters in SPF minipigs

**Figure 1:**
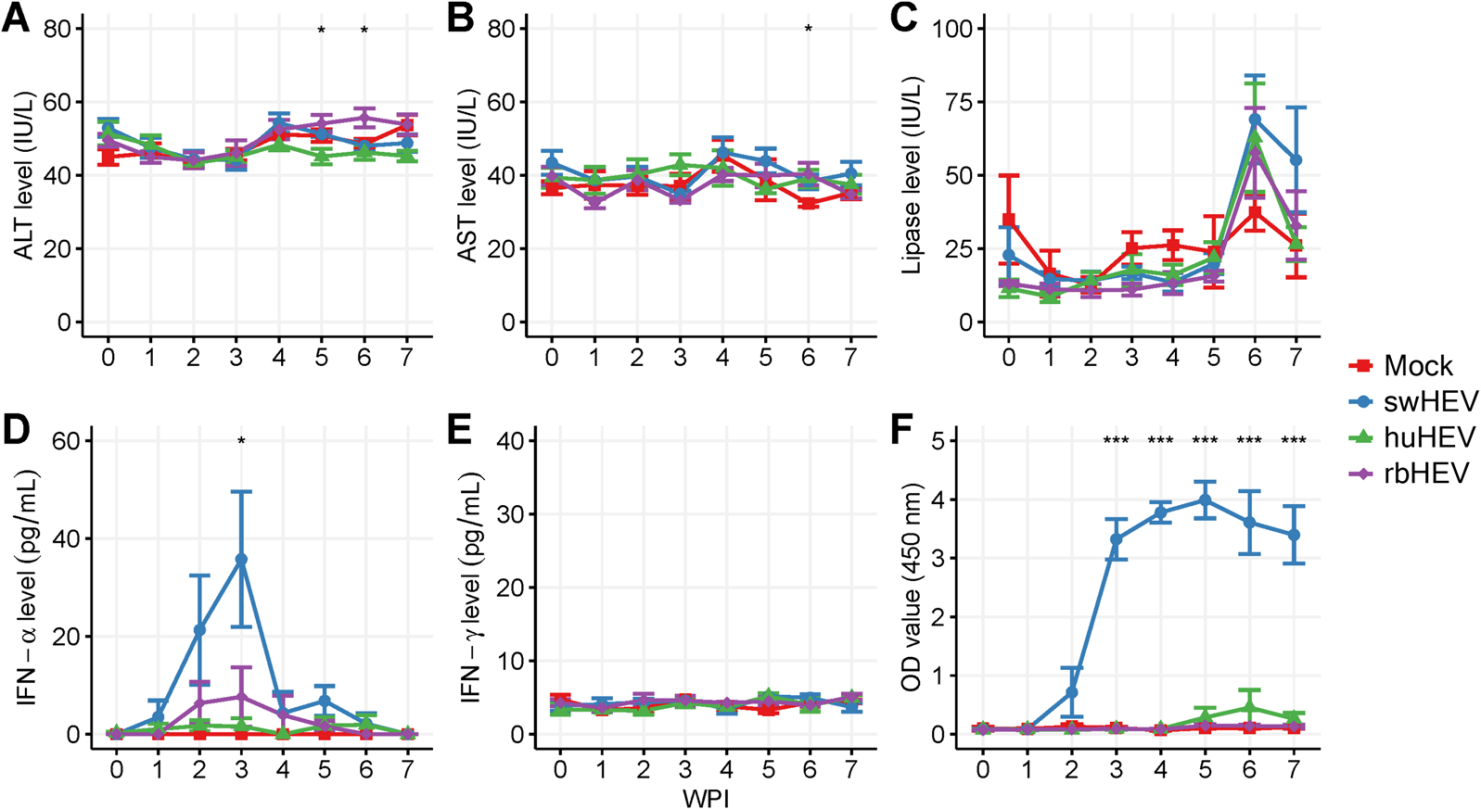
**Time course of serological indicators in minipigs infected with HEV from different species**. All experiments were performed using plasma samples. **A** ALT levels. The normal range was 31–58 IU/L. **B** AST levels. The normal range was 32–84 IU/L. **C** Lipase levels. The normal range was < 100 IU/L. **D** IFN-α levels, **E** IFN-γ levels, and **F** seroconversion. The cut-off value was calculated as 0.5 plus the mean absorbance of the non-reactive control. *p < 0.05, ***p < 0.001

Acute hepatitis caused by HEV results in notable serological changes, with ALT and AST levels serving as representative indicators of liver damage caused by the virus. Accordingly, the ALT and AST levels were analysed in the experimental groups, along with the lipase levels, as an indicator of pancreatic damage. Until the end of the test at 7 wpi, both ALT and AST levels remained flat, with no significant change in any of the groups (Figures 1A and B). A significant increase in ALT levels was observed in the huHEV and rbHEV groups compared with the mock group at 5 wpi (*p* = 0.020) and 6 wpi (*p* = 0.025), respectively; however, the ALT levels were within the normal range (31–58 IU/L). Similarly, at 6 wpi, all three HEV groups showed significantly higher AST levels than the mock group (*p* < 0.05), but the values were within the normal range (32–84 IU/L). In the three HEV-inoculated groups, the lipase concentration remained low until 5 wpi and then climbed steeply at 6 wpi (swHEV, 59.1 ± 15.5; huHEV, 62.8 ± 16.0; rbHEV 57.7 ± 1.3 IU/L) (Figure 1C). By 7 wpi, the lipase levels in the huHEV and rbHEV groups had decreased suddenly (26.5 ± 5.0 and 32.9 ± 10.1 IU/L, respectively), but those in the swHEV group remained consistent (55.2 ± 15.5 IU/L). While the mock group also showed a slight increase in lipase levels at 6 wpi (37.4 ± 5.1), this difference was not statistically significant.

The levels of type I and II IFNs, which are representative antiviral cytokines, were measured (Figures 1D and E). Moreover, anti-HEV antibodies were detected as immunological indicators of HEV infection (Figure 1F). The levels of IFN-α, a type I IFN, increased significantly (*p* < 0.05) in the swHEV group beginning at 1 wpi (3.498 ± 3.40 pg/mL) and peaked at 3 wpi (35.8 ± 12.0 pg/mL). At 3 wpi, the IFN-α level suddenly decreased and was not detected at 7 wpi. The IFN-α levels in the rbHEV group were similar to those in the swHEV group but with lower values. In the rbHEV group, the IFN-α level gradually increased beginning at 2 wpi, peaked at 3 wpi (7.6 ± 6.1 pg/mL), and then gradually decreased. In the huHEV and mock groups, IFN-α was either not detected or detected at a very low concentration. IFN-γ, a type II interferon, was detected only at low levels in all four groups. In the swHEV group, the levels of HEV-specific antibodies increased steeply from 2 wpi and reached a plateau starting at 3 wpi (*p* < 0.001, compared to the mock group). No HEV-specific antibodies were detected in the rbHEV or mock groups, and in the huHEV group, HEV-specific antibody levels slightly increased from 5 to 6 wpi and then decreased to a level similar to that in the mock group by 7 wpi.

### Different HEV strains cause different histological changes in tissues

**Figure 2:**
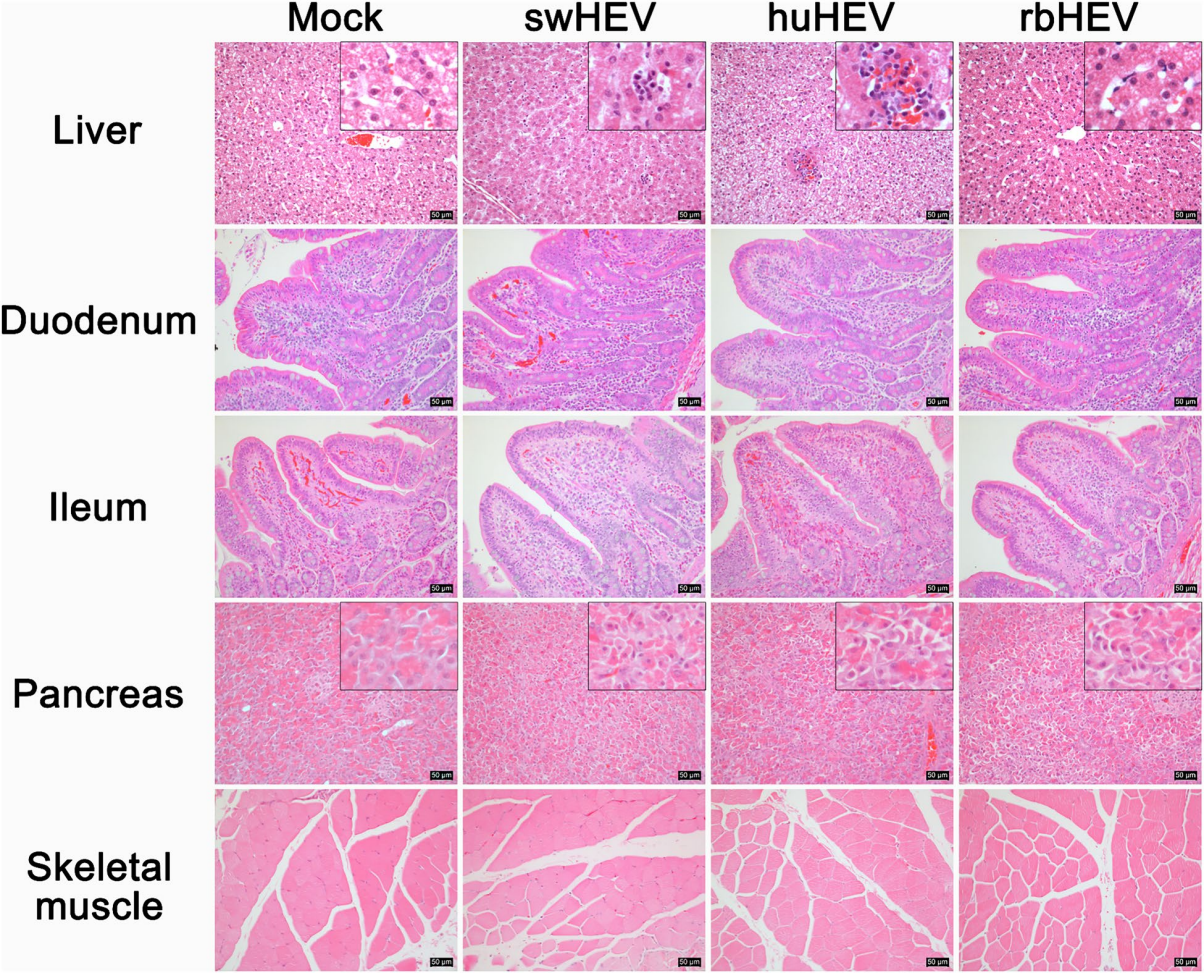
**Histopathological alterations in the major tissues of minipigs infected with HEV from different species.** The tissues were photographed at 100 × magnification, with the enlarged image shown at 400 × magnification. Scale bars = 50 μm

The liver, the main target organ of HEV, showed different histological changes depending on the HEV strain (Figure 2, row 1). In the livers of the swHEV group, Mallory’s hyaline changes with mild microvesicular steatosis and Kupffer cell hyperplasia were observed in the anterior lobe of the liver, and focal inflammatory cell infiltration and single-cell apoptosis were observed. In the huHEV group, the livers exhibited pan-lobular hydropic degeneration, and slightly more severe focal inflammatory cell infiltration than that in the swHEV group was observed. No histopathological changes were observed in the rbHEV or mock groups. Simultaneously, the intestine showed relatively common pathological changes without differences in strain (Figure 2, rows 2, 3). In the duodenum, plasma cells and eosinophils were observed in the lamina propria in all three HEV groups, especially in the swHEV group. In the ileum, thickening of the villi due to increased immune cell numbers in the lamina propria was observed in all three HEV groups. In the pancreas, histological changes, such as pyknosis and karyorrhexis, and accompanied by cell membrane rupture, were broadly observed in acinar cells in all three HEV groups, despite the lack of significant differences in the levels of blood biochemical indicators compared with those in the mock group (Figure 2, row 4). Conversely, no histopathological alterations in skeletal muscle were detected in any group (Figure 2, row 5).

**Figure 3:**
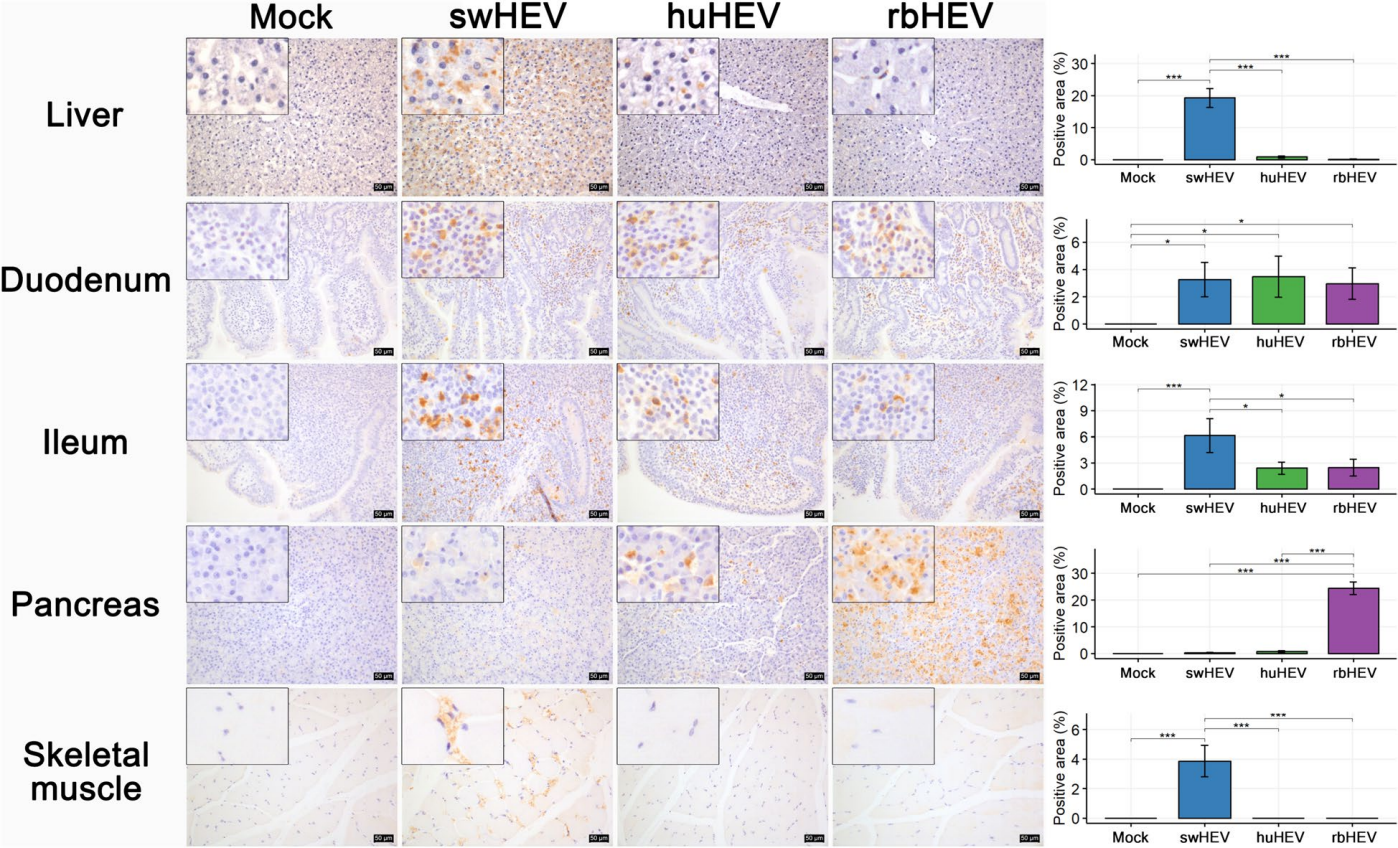
**HEV antigen localization in major tissues of minipigs infected with HEV from different species.** The brown color represents the HEV antigen; nuclei were counterstained with Meyer’s hematoxylin. The bar plot shows the HEV antigen-positive area/total area (%). The tissues were photographed at 100 × magnification, with the enlarged image shown at 400 × magnification. Scale bars = 50 μm. *p < 0.05, **p < 0.01, ***p < 0.001

**Figure 4:**
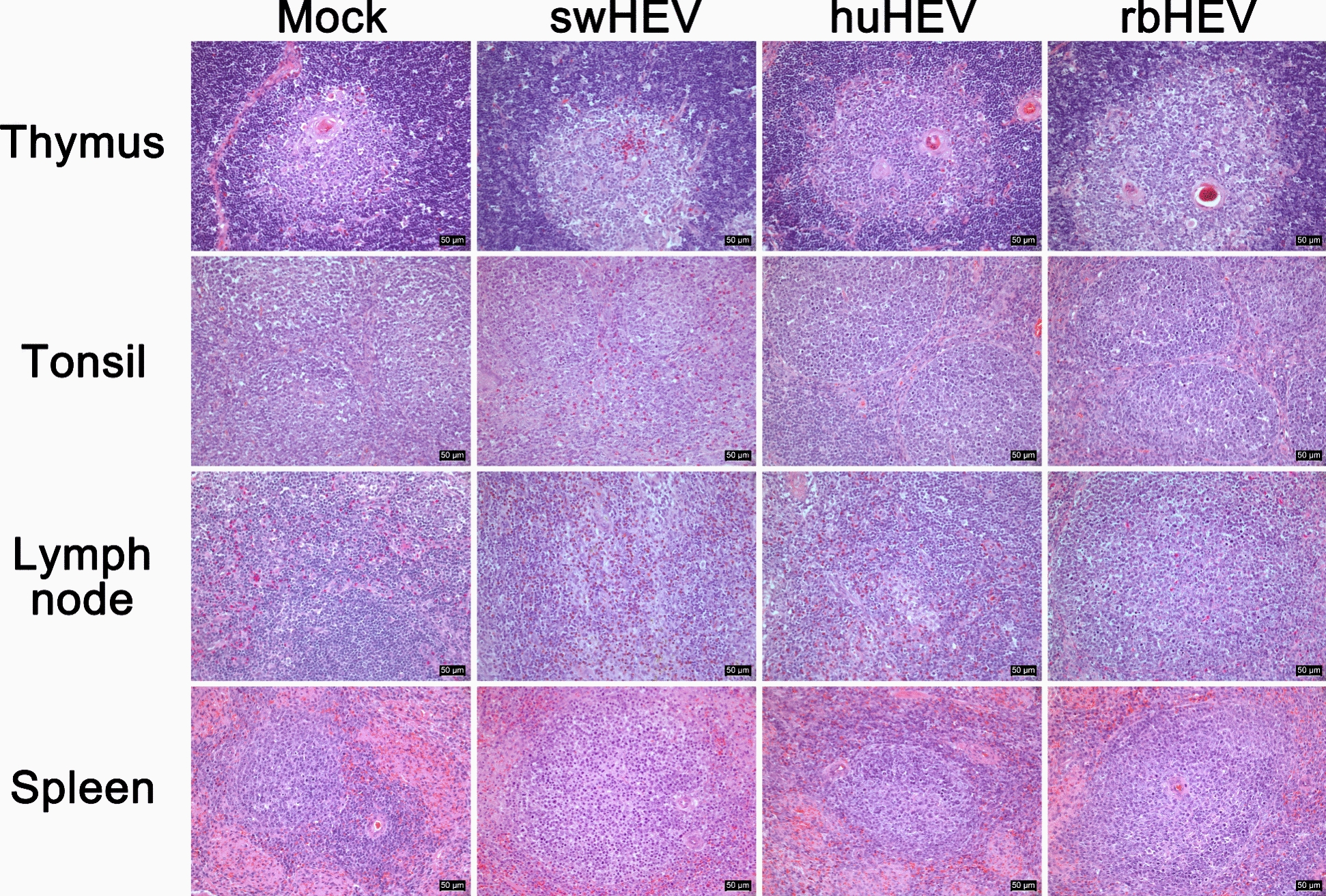
**Histopathological alterations in the lymphoid tissues of minipigs infected with HEV from different species**. The tissues were photographed at 100 × magnification, with the enlarged image shown at 400 × magnification. Scale bars = 50 μm

The distribution of different HEV strains within the liver resembled the pattern of liver histopathological changes (Figure 3, row 1). In the swHEV group, swHEV was observed in the perinuclear and cytoplasmic regions of hepatocytes and was extensively distributed across the entire lobe. Like swHEV, huHEV was observed in the cytoplasm of hepatocytes but to a lesser extent. On the other hand, rbHEV was not detected in hepatocytes and was detected only in some Kupffer cells. In the intestine, all three strains of HEV exhibited nearly identical distribution patterns (Figure 3, rows 2, 3). HEVs were not detected in enterocytes and were predominantly distributed in immune cells within the lamina propria. Among the three strains, swHEV exhibited the highest signal strength. HEV was heavily distributed throughout the pancreas. (Figure 3, row 4). Low-intensity swHEV signal was detected in the cytoplasm of pyknotic and karyorrhectic acinar cells. huHEV was observed to have a stronger signal intensity in karyorrhectic acinar cells and a weaker signal intensity in the cytoplasm of normal acinar cells. In contrast, rbHEV was distributed across the entire pancreatic lobe, irrespective of the cell type; however, its histological distribution was similar to that of other HEVs. None of the three HEV strains were detected within the Langerhans islets. In the skeletal muscle, only swHEV was distributed in the endomysium (Figure 3, row 5) (see Figure 4).

**Figure 5:**
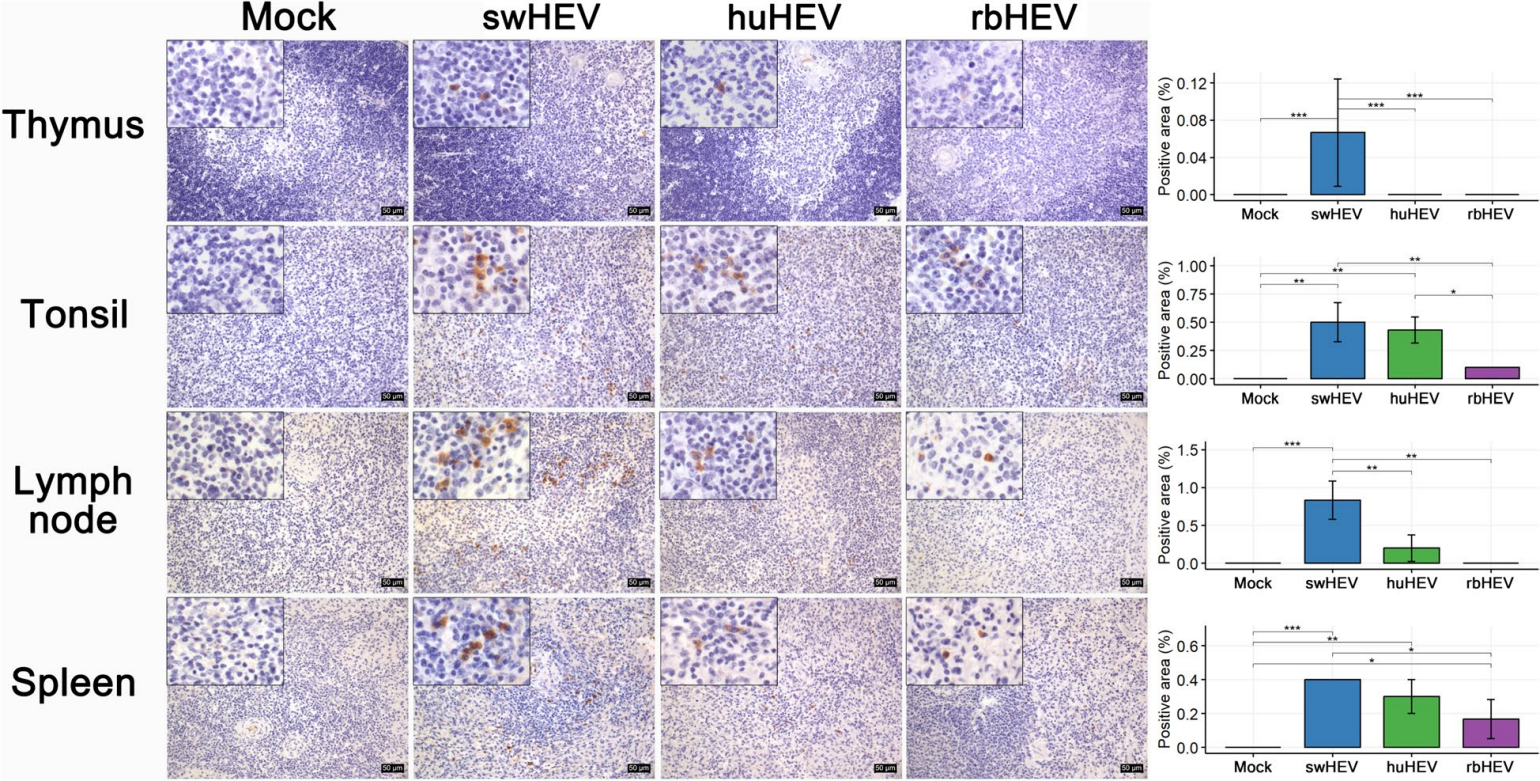
**HEV antigen localization in the lymphoid tissues of minipigs infected with HEV from different species**. The brown color represents the HEV antigen; nuclei were counterstained with Meyer’s hematoxylin. The bar plot shows the HEV antigen-positive area/total area (%). The tissues were photographed at 100 × magnification, with the enlarged image shown at 400 × magnification. Scale bars = 50 μm. *p < 0.05, **p < 0.01, ***p < 0.001

The histopathological alterations observed in the lymphoid organs were less severe than those observed in the liver and pancreas. In the thymus, eosinophil infiltration was noted within the medullary region in three HEV groups, with the most pronounced increase in the swHEV group (Figure 4, row 1). In the huHEV group, apoptotic cells characterized by eosinophilic cytoplasm and condensed nuclei were identified within the medulla. In the tonsils, the swHEV group exhibited diffuse paracortical hyperplasia accompanied by eosinophilia, whereas the huHEV and rbHEV groups displayed numerous mitotic cells in the follicles due to reactive follicular hyperplasia (Figure 4, row 2). The histopathological changes in the lymph nodes were similar to those observed in the tonsils (Figure 4, row 3). Diffuse paracortical hyperplasia accompanied by eosinophilia was observed in both the swHEV and huHEV groups. Additionally, hemosiderin pigment was observed in the swHEV group. In the rbHEV group, more pronounced follicular hyperplasia was observed than in the tonsils. In the spleen, follicular hyperplasia was observed in all three groups, with a notable presence of mitotic cells throughout the follicles, particularly in the swHEV group (Figure 4, row 4). HEV distribution was noted in all lymphoid organs, albeit to a lesser degree than that in the parenchymal organs. In the thymus, each of the three HEVs was observed in a small number of cells within the medulla (Figure 5, row 1). In the tonsils, lymph nodes, and spleen, HEV was sporadically observed in the follicles and paracortex, with the highest frequency being observed in the swHEV group, followed by the huHEV group, and finally, the rbHEV group (Figure 5, rows 2–4).

## Discussion

The purpose of the present study was to evaluate the infectivity of HEV from different species in mini-pigs and to compare the infection patterns and histopathological characteristics. The present study demonstrated that three strains of HEV genotype 3 (swine, rabbit, and human) can infect minipigs, as indicated by tissue-specific histopathological changes and the distribution of HEV. All three HEVs were distributed in infected immune cells in the small intestine and immune organs, and strain-specific distribution patterns were observed in the liver, pancreas, and muscle. However, fecal shedding and viremia were strongly apparent after swHEV infection in weeks 1–4, whereas huHEV and rbHEV were detected intermittently. This difference may be related to the influence of interspecies barriers affecting the susceptibility of pigs to swHEV and the species specificity of the virus strains. In studies where rabbits were infected with swHEV and rbHEV, fecal shedding and viremia were observed in swHEV-infected rabbits approximately 1–2 weeks later than in rbHEV-infected rabbits [23, 25]. Moreover, studies involving conventional domestic pigs have also demonstrated that swHEV leads to fecal shedding and viremia within 1–2 weeks, whereas rbHEV shedding and viremia occur within 5–7 weeks, indicating a delay of more than 4 weeks [23, 26]. This interspecies barrier may be attributed to viral factors, including the open reading frame 1 of HEV, adaptive evolution, and codon usage, or host factors, such as cellular characteristics and host immune status [27]. However, in the aforementioned studies, HEV from other species also influenced serological indicators, such as seroconversion and the levels of certain proinflammatory cytokines. In contrast, in the present study, only swHEV affected serological indicators in minipigs. This discrepancy may be attributed to intrastrain differences. According to a study by Li et al. [28], even if HEV originates from the same species, the kinetics of infection indicators, such as fecal shedding, viremia, and seroconversion, differ depending on the genotype/subtype.

In various animal models of HEV infection, histopathological changes are frequently more sensitive indicators than virological and serological signs. For example, histopathological changes in the liver are more sensitive in most studies than elevations in ALT and AST levels [25, 29, 30]. Likewise, in this study, although no elevation in the levels of liver enzymes was observed, different strains had different pathological effects on the liver tissues. swHEV was distributed throughout the liver lobe, with Mallory’s hyaline bodies and microvesicular steatosis observed. huHEV was localized to a small area of the hepatocyte and was accompanied by hydropic changes, whereas rbHEV was found only in Kupffer cells and was not associated with any tissue changes. These differences between strains may be related to differences in the pathogenicity of each strain or, as mentioned above, to the delay in infection due to the interspecies barrier. According to a previous study that revealed histopathological changes over time in HEV-infected minipigs, extensive inflammatory cell infiltration was observed in the liver at 3 wpi, and HEV antigen began to be detected in hepatocytes at the time the infiltrating cells disappeared [29]. Over the seven weeks of this study, a consistent increase in the amount of HEV antigen was observed in hepatocytes, suggesting that hepatitis symptoms (elevated ALT levels) caused by HEV may appear after more than seven weeks. The 47832c strain used as a huHEV was isolated from the serum of a kidney transplant patient with chronic hepatitis E and has been adapted for use in cell culture [31]. Chronic hepatitis E infection is usually associated with lower ALT elevation than acute hepatitis E infection [3]. Additionally, the 47832c strain may have been attenuated during multiple subcultures using cells. Therefore, the huHEV strains used in the present study may be attenuated compared to the HEV strains that cause acute hepatitis. In a chronic HEV infection experiment using immunocompromised monkeys, liver lesions developed from hydropic changes in inflammatory cell infiltration at 69 dpi, which was slower than the development of acute hepatitis E lesions in monkeys (approximately 25 dpi) [32, 33]. In this study, the huHEV utilized may exist in a quasi-enveloped form, as it was obtained from supernatant cultured in a cell line. The entry of quasi-enveloped HEVs requires additional lysosomal degradation of the viral membrane compared to that of non-enveloped HEVs [7]. Considering these characteristics of chronic hepatitis E and the time course of histopathological data [29], infection with huHEV may occur approximately five weeks later than infection with swHEV. On the other hand, although rbHEV is derived from a special model of pregnant rabbits, it is presumed to be highly pathogenic because it causes the localization of multiple antigens to hepatocytes, elevated ALT levels, and high rates of stillbirth at only 14 dpi [34]. Therefore, the delay in infection with rbHEV may be due to the interspecies barrier, as is the case with serological indicators. Likewise, when considering time course histopathological data [29], rbHEV may have delayed infection by more than 7 weeks compared to swHEV.

Acute pancreatitis is a well-known phenomenon in both HEV and other types of viral hepatitis. Regarding HEV, cases of acute pancreatitis have been reported for genotypes 1, 3, and 4, with cases of genotypes 3 and 4 being partially corroborated in animal models [29, 35, 36]. In the present study, histopathological changes in the pancreas were similar across the three HEV groups, and the pancreatic localization of rbHEV was more prominent than that of swHEV. Given these results, the association of HEV with pancreatitis may be a general manifestation that is not limited to specific strains, susceptible hosts, or viruses. Additionally, these findings suggest that the pancreas may be the initial site of HEV replication before it reaches the liver. Similarly, Jung et al. [29] reported that swHEV was extensively localized in the pancreas of minipigs at 21 dpi, 7 d before it was detected in the cytoplasm of hepatocytes. Therefore, rbHEV, which showed extensive localization in the pancreas at 7 wpi, may cause infection in minipigs approximately four weeks later than swHEV. However, the localization of HEVs to the pancreas before the liver may be an issue specific to minipigs. In a study in which conventional pigs were inoculated with swHEV and observed up to 55 dpi, HEV RNA was not detected in the pancreas until the end of the experiment [37].

In the present study, compared with swHEV, rbHEV and huHEV were thought to exhibit delayed infection but showed similar localization, i.e., localization to the small intestine and lymphoid tissues, accompanied by mild histopathological changes. The small intestine (duodenum, ileum) and lymphoid tissue (thymus, tonsils, lymph nodes, and spleen) are the tissues in which HEVs are most commonly found after the liver [29, 37]. According to a study by Sayed et al. [38], HEV not only survives and persists in human monocytes, macrophages, and bone marrow-derived macrophages but also completes its entire life cycle inside immune cells and is capable of replication. Moreover, PBMCs may be a reservoir for HEV and a source of extrahepatic manifestations. Therefore, HEV may be distributed to various organs other than the liver and pancreas through infected immune cells, and the interspecies barrier may not apply to HEV distribution through these immune cells. Future studies are needed to gain further insights into how the distribution of HEV across immune cells transcends the interspecies barrier. To the best of our knowledge, this study confirmed, for the first time, the presence of HEV antigen in the skeletal muscle of experimental animals. Myositis due to HEV has been reported in immunocompetent patients and liver transplant recipients, with patients suffering from severe muscle weakness [39, 40]. Muscle biopsies revealed scattered myofiber necrosis accompanied by diffuse mild lymphomonocytic infiltrate but did not confirm the presence of HEV. Although necrosis and cellular infiltration were not observed in muscle tissue in the present study, it is possible that HEV directly causes myositis. A limitation of the current study is that although there were differences in the histological changes and distribution of HEV in various organs in minipigs infected with HEV from different species, these differences were not directly linked to clinical symptoms or serological factors such as plasma ALT and lipase. Additionally, this study suggested that HEV can be transferred to various organs through infected immune cells regardless of the strain, but this may have occurred because the virus was administered intravenously in this study. Further studies will determine whether orally infected HEVs cause the same phenomenon in the pancreas and immune organs.

In conclusion, the present study demonstrated that HEV genotype 3 from three different species (swine, human, and rabbit) infects minipigs, is distributed to different organs, such as the liver and pancreas, and causes histopathological alterations. Additionally, based on virological, serological, and histological analyses, compared to infection with swHEV, infection was delayed by approximately 5 weeks for huHEV and 4–7 weeks or more for rbHEV. This delay in infection may be due to interspecies barriers or the pathogenicity of the strain. This suggests that pigs may be an intermediate host for the transmission of HEV from various species to humans.

## Data Availability

All data underlying the results are available as the article and no additional source data are required.
